# Nutrient Enrichment Increases Grassland Biomass Sensitivity to Interannual Precipitation Variation

**DOI:** 10.1111/gcb.71053

**Published:** 2026-08-17

**Authors:** Ingrid J. Slette, Eric W. Seabloom, Carlos Alberto Arnillas, Jonathan D. Bakker, Dana Blumenthal, Jane A. Catford, Scott L. Collins, Nico Eisenhauer, Philip A. Fay, Magda Garbowski, Sylvia Haider, W. Stanley Harpole, Kevin P. Kirkman, Johannes M. H. Knops, Kimberly J. Komatsu, Andrew D. B. Leakey, Andrew S. MacDougall, Jason P. Martina, Sally A. Power, Anita C. Risch, Christiane Roscher, Melinda D. Smith, Michelle Tedder, Elizabeth T. Borer

**Affiliations:** ^1^ Department of Ecology, Evolution and Behavior University of Minnesota St. Paul Minnesota USA; ^2^ Department of Physical and Environmental Sciences University of Toronto at Scarborough Toronto Ontario Canada; ^3^ School of Environmental and Forest Sciences University of Washington Seattle Washington USA; ^4^ USDA‐ARS Rangeland Resources & Systems Research Fort Collins Colorado USA (Emeritus); ^5^ Department of Geography King's College London London England; ^6^ Fenner School of Environment & Society The Australian National University Canberra Australia; ^7^ Department of Biology University of New Mexico Albuquerque New Mexico USA; ^8^ German Centre for Integrative Biodiversity Research (iDiv) Halle‐Jena‐Leipzig Leipzig Germany; ^9^ Institute of Biology Leipzig University Leipzig Germany; ^10^ USDA‐ARS Grassland, Soil, and Water Lab Temple Texas USA; ^11^ Animal and Range Sciences New Mexico State University Las Cruces New Mexico USA; ^12^ Institute of Ecology Leuphana University of Lüneburg Lüneburg Germany; ^13^ Department of Physiological Diversity Helmholtz Center for Environmental Research–UFZ Leipzig Germany; ^14^ Martin Luther University Halle‐Wittenberg Halle (Saale) Germany; ^15^ School of Agriculture and Science University of KwaZulu‐Natal Pietermaritzburg South Africa; ^16^ School of Biological Sciences University of Nebraska Lincoln Nebraska USA; ^17^ Department of Biology University of North Carolina at Greensboro Greensboro North Carolina USA; ^18^ Department of Plant Biology University of Illinois Urbana‐Champaign Urbana Illinois USA; ^19^ Department of Integrative Biology University of Guelph Guelph Ontario Canada; ^20^ Department of Biology Texas State University San Marcos Texas USA; ^21^ Hawkesbury Institute for the Environment Western Sydney University Penrith New South Wales Australia; ^22^ Swiss Federal Institute for Forest Snow and Landscape Research WSL Birmensdorf Switzerland; ^23^ Department of Biology and Graduate Degree Program in Ecology Colorado State University Fort Collins Colorado USA

**Keywords:** aridity, biomass, correlation strength, grassland, interannual variability, nutrient addition, Nutrient Network (NutNet), precipitation, precipitation sensitivity, rain use efficiency

## Abstract

Primary production underpins much of ecosystem functioning and is often limited by nutrient and water availability. Ongoing global increases in nutrient inputs and interannual precipitation variability are thus likely to impact primary production, with unclear consequences for interannual variability in plant biomass. We used data from 35 grasslands in the Nutrient Network coordinated fertilization experiment to test if nutrient addition alters the response of plant biomass to year‐to‐year variation in growing season precipitation, and whether the effects of nutrient addition vary with factors such as site aridity or plant community composition. Nutrient addition increased biomass (by an average of 46% across sites), rain‐use efficiency (by 48%), and the sensitivity of biomass to interannual variation in precipitation (i.e., larger increase in biomass in wet years; by 13%), but did not change the strength of the interannual precipitation‐biomass correlation. Both sensitivity and correlation strength were higher in more arid sites, regardless of nutrient addition. These results suggest that grassland ecosystem functioning may become more variable from year to year as nutrient enrichment increases biomass sensitivity to precipitation and precipitation becomes more variable.

## Graphical abstract

We used data from 35 grasslands in the Nutrient Network (NutNet) to test if NPK addition alters how plant biomass responds to year‐to‐year variation in precipitation. We found that nutrient addition increased average biomass, rain‐use efficiency, and the sensitivity of biomass to interannual variation in precipitation, but did not change the strength of the interannual precipitation‐biomass correlation. Interannual biomass variability will likely increase as nutrient inputs and precipitation variability increase globally.

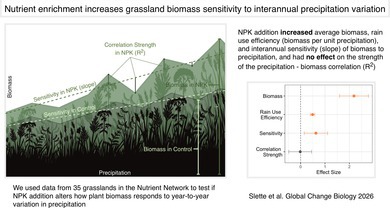

## 1 Introduction

Primary production is foundational to ecosystem functioning and provision of services (habitat, forage, fuel, etc.) and is an essential component of the global carbon cycle (Cardinale et al. 2012; Gounand et al. 2020; Kremen 2005). Because primary production is often limited by nutrient and water availability (Elser et al. 2007; Fay et al. 2015; Harpole et al. 2011; Huxman et al. 2004; Knapp and Smith 2001; Lauenroth and Sala 1992; LeBauer and Treseder 2008; Sala et al. 1988), ongoing increases in nutrient inputs and precipitation variability globally (Douville et al. 2021; Giorgi et al. 2019; Pfahl et al. 2017; Rockström et al. 2009; Stevens et al. 2004; Zhang et al. 2021) are likely to alter primary production. How this will affect interannual (i.e., year‐to‐year) variability in plant biomass remains unclear. It is well established that mean annual aboveground grassland biomass increases with increasing mean annual precipitation (MAP; Sala et al. 1988) and that the interannual precipitation‐biomass relationship within individual sites is consistently weaker and more variable than the long‐term average relationship among sites (Huxman et al. 2004; Maurer et al. 2020; Sala et al. 2012). It has also recently been found that nutrient addition increases long‐term average biomass sensitivity to MAP (Fay et al. 2025). However, the impact of nutrient addition on the interannual precipitation‐biomass relationship remains less clear, as the methods and results of studies on this topic differ (e.g., regional experiments by Collins et al. 2025 and Morgan et al. 2016; literature review by Hooper and Johnson 1999; meta‐analysis by Yahdjian et al. 2011). We thus lack a generalized understanding of how nutrient addition affects the interannual relationship between precipitation and biomass, and what factors explain different responses among sites.

In herbaceous‐dominated ecosystems (hereafter, “grasslands”), evaporative demand typically exceeds precipitation for at least part of the growing season. Consequently, grassland primary production is usually water‐limited and sensitive to precipitation change (Huxman et al. 2004; Knapp and Smith 2001; Lauenroth and Sala 1992; Sala et al. 1988). This sensitivity and their vast global extent make grasslands important systems for studying precipitation‐production relationships (Epstein et al. 2006; MacDougall et al. 2026; White et al. 2000). Though average grassland biomass is strongly correlated with average precipitation over large spatial scales and long temporal scales (Fay et al. 2025; Sala et al. 1988; MacDougall et al. 2024), interannual fluctuations in grassland biomass are often only weakly related to interannual fluctuations in precipitation within a site (Huxman et al. 2004; Maurer et al. 2020; Sala et al. 2012; Wang and Collins 2024). Many possible explanations have been proposed for why current‐year biomass is not more strongly correlated with current‐year precipitation. One possibility is that biomass responsiveness to precipitation is constrained by nutrient availability. Plants need nutrients as well as water, and nutrient addition often increases grassland biomass (Carroll et al. 2025; Fay et al. 2025; Li et al. 2024). Assuming co‐limitation by water and nutrients, we expect that reducing nutrient limitation via fertilization could make interannual variation in biomass more sensitive to, and more strongly correlated with, interannual variation in precipitation. If biomass and precipitation indeed become more tightly coupled when nutrient availability increases, biomass would likely become more variable from year to year, as ongoing changes to the global environment increase both nutrient inputs and interannual precipitation variability.

**FIGURE 1:**
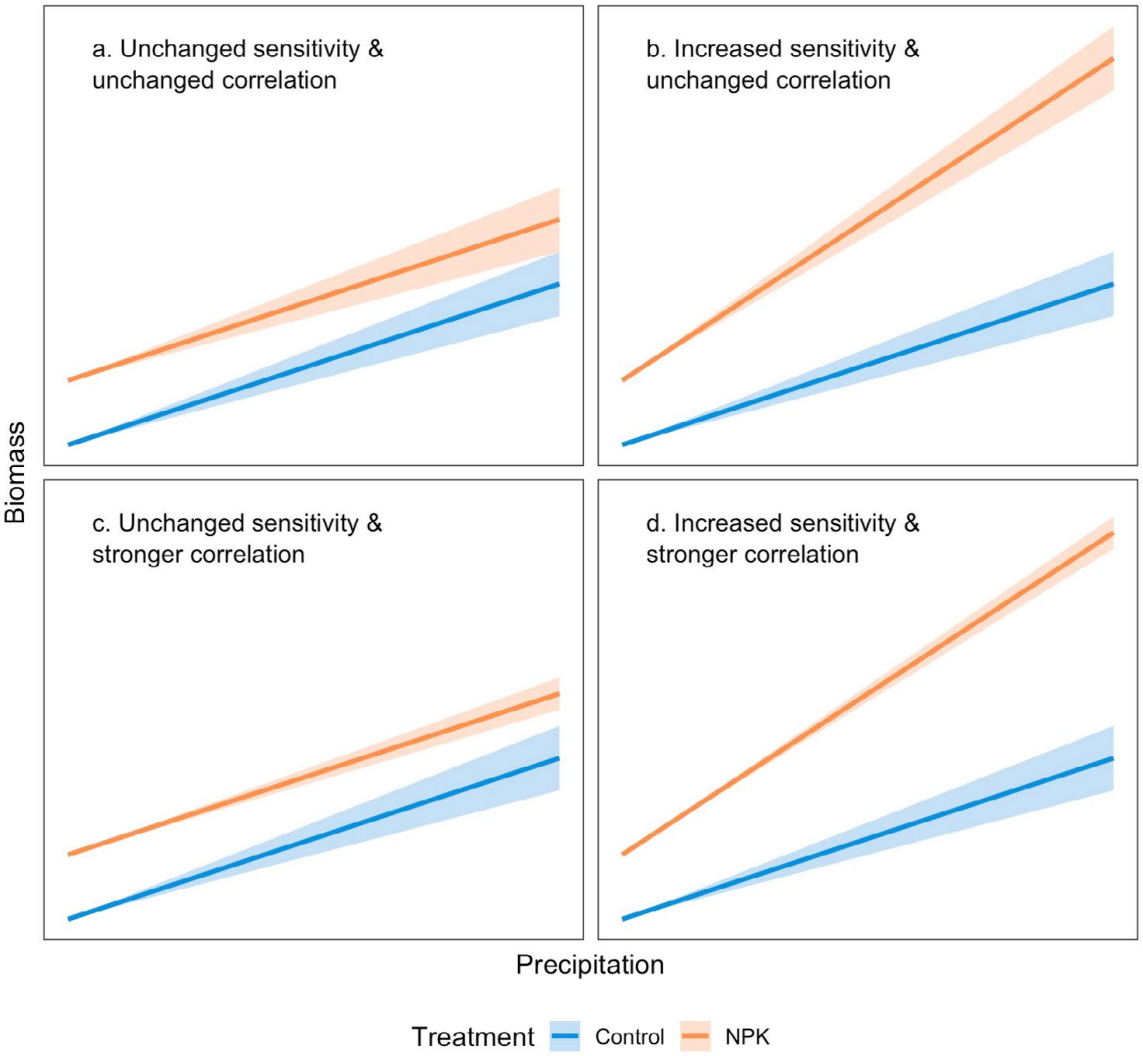
Conceptual representation of how nutrient addition could affect the interannual precipitation‐biomass relationship. (a) Adding nutrients could increase biomass independent of precipitation, such that biomass increases equally at all precipitation levels, with no change in biomass sensitivity to precipitation or the strength of the precipitation‐biomass correlation. (b) Alternatively, adding nutrients could interact with precipitation to increase biomass sensitivity, such that fertilization increases biomass more during wetter years. More biomass at the same level of precipitation in fertilized versus control plots would indicate higher rain‐use efficiency (RUE; biomass/precipitation). By reducing nutrient limitation, adding nutrients could make production more tightly coupled to other limiting factors such as water, thus strengthening the correlation between precipitation and biomass, either without (c) or with (d) also increasing biomass sensitivity to precipitation.

Adding nutrients could increase biomass independent of precipitation amount, such that biomass increases equally at all precipitation levels, with no change in biomass sensitivity to precipitation or the strength of the precipitation‐biomass correlation (Figure 1a). Alternatively, adding nutrients could interact with precipitation amount to increase biomass sensitivity, such that fertilization increases biomass more during wetter years, when water is less limiting (Figure 1b). More biomass at the same level of precipitation in fertilized versus control plots would indicate higher rain‐use efficiency (RUE; biomass/precipitation), suggesting a more efficient conversion of water into plant biomass with nutrient enrichment. Reducing nutrient limitation via fertilization might also make production more tightly coupled to remaining limiting factors such as water. Adding nutrients could thus strengthen the correlation between precipitation and biomass, either without (Figure 1c) or with (Figure 1d) also increasing biomass sensitivity to precipitation. For this study, we define sensitivity as the change in biomass per change in precipitation, and we define correlation strength as the amount of variability in biomass explained by variability in precipitation. We quantify sensitivity and correlation strength as the slope and the *R* ^2^ of the interannual precipitation‐biomass relationship, respectively.

In addition to water and nutrient availability, other biotic and abiotic variables can also influence plant biomass production and the precipitation‐biomass relationship. For example, biomass sensitivity to precipitation does not vary randomly among sites but is typically higher in more arid sites (i.e., the “Huxman‐Smith model”; Hedberg et al. 2025; Huxman et al. 2004; Knapp et al. 2015, 2024; Maurer et al. 2020). Biomass can also be affected by previous‐year precipitation in addition to current‐year precipitation (i.e., “legacy effects”; He et al. 2025; Goodman and Felton 2025; Griffin‐Nolan et al. 2018; Sala et al. 2012). And because plant growth requires light as well as water and nutrients, changes in water and nutrient supply might have a smaller effect on biomass at sites where light limits production due to high biomass reducing light below the plant canopy than at sites where light is plentiful through all vertical canopy layers (Farrior et al. 2013). Changes in plant community composition following nutrient addition can also alter plant productivity (Avolio et al. 2014). For example, fertilization often decreases species richness (Hillebrand et al. 2007; Isbell et al. 2013), and low richness can amplify the negative effect of dry years on plant production (Isbell et al. 2015; Tilman and Downing 1994). Plant traits could also affect the precipitation‐biomass relationship. Plants with an annual lifespan might be more sensitive to interannual precipitation variability than perennial plants, as annual plants can respond to altered resource availability on shorter timescales (Cleland et al. 2013; Wilcox et al. 2021). Species with the C_4_ photosynthetic pathway might be less sensitive to interannual precipitation variability than species with the C_3_ photosynthetic pathway, as C_4_ species generally have higher water‐use efficiency than C_3_ species (Monson et al. 2025). Further, the effects of nutrient enrichment on biomass might increase over time (Seabloom et al. 2021). The degree of nutrient limitation at a site can also affect how fertilization alters the precipitation‐biomass relationship in the long term (Fay et al. 2015, 2025). Herbivory intensity can also alter the precipitation‐biomass relationship to varying degrees in different grasslands (Irisarri et al. 2016). We expect the interannual precipitation‐biomass relationship to vary among sites, with differences in the factors listed above helping to explain site specificity in the interannual precipitation‐biomass relationship and how it changes with nutrient addition.

**FIGURE 2:**
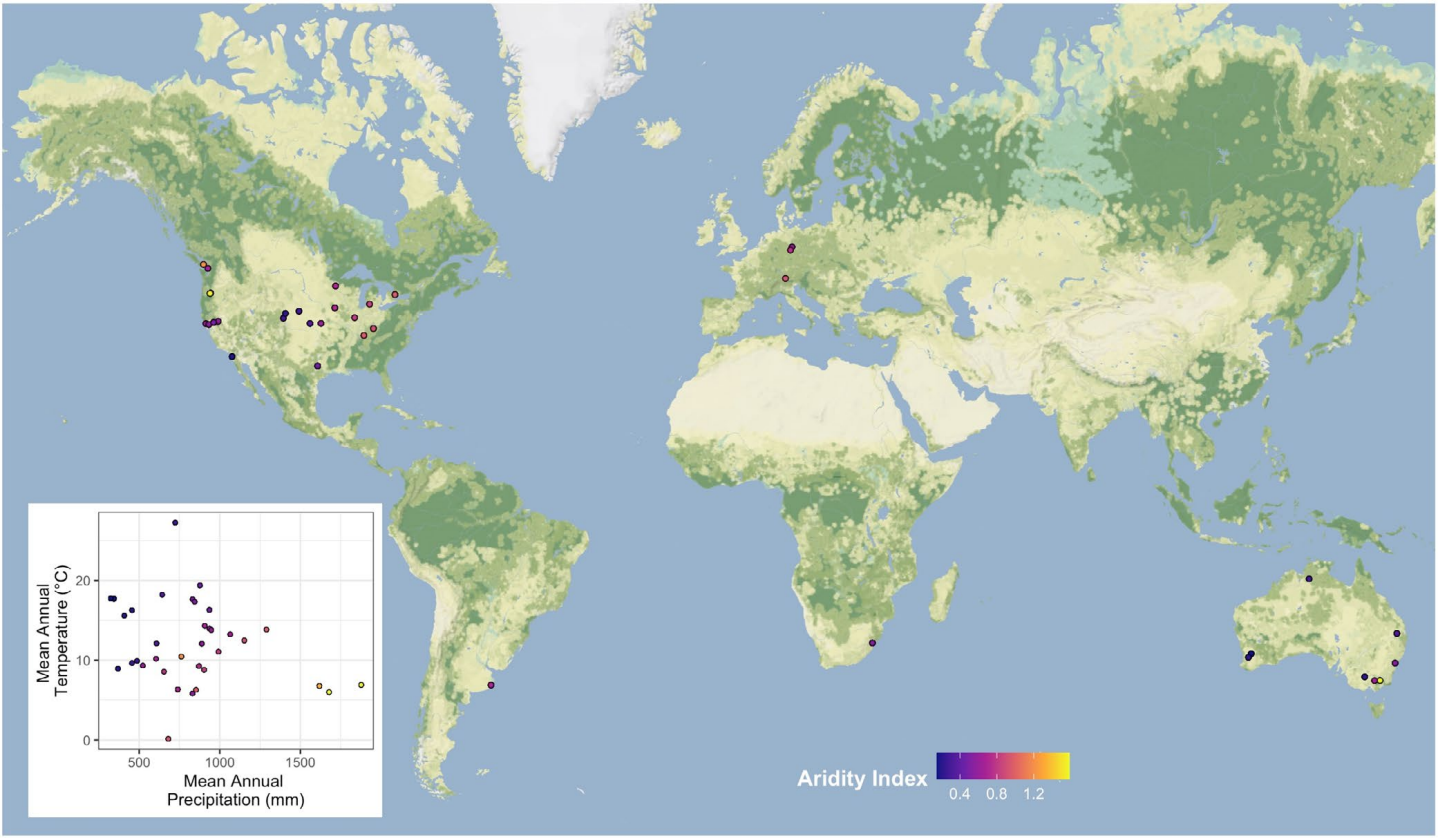
Site locations (*n* = 35), mean annual precipitation, and mean annual temperature, colored by aridity index (lower aridity index values = more arid).

We assessed how nutrient addition affects the interannual relationship between growing season precipitation and aboveground plant biomass using data from 35 grassland sites located across Africa, Australia, Europe, and North and South America (Figure 2), with more than 5 years of data from both fertilized and unfertilized plots from the Nutrient Network experiment (NutNet; Borer et al. 2014) and a range of annual precipitation amounts representative of the site‐specific long‐term record. We then tested whether additional factors (site aridity, previous growing season precipitation, light interception, plant species richness, proportional annual species cover, proportional C_4_ species cover, number of treatment years, and degree of site nutrient limitation) affected the interannual precipitation‐biomass relationship and how it changed with nutrient addition. We hypothesized that nutrient addition would increase average biomass, rain‐use efficiency, sensitivity of biomass to precipitation, and the strength of the precipitation‐biomass correlation across all sites. We further hypothesized that responses to nutrient addition would vary among sites, with differences in the additional co‐variates listed above helping to explain that variation.

## 2 Methods

### 2.1 Experimental Design

We used data from the Nutrient Network (NutNet) coordinated distributed experiment, a globally replicated nutrient and herbivore manipulation experiment (Borer et al. 2014). We included NutNet sites with more than 5 years of data (range: 6–16 years) from both fertilized (NPKμ) and unfertilized (control) plots and a growing season precipitation range representative of the long‐term site record (> ±1 standard deviation from average—details below). The complete NutNet protocol can be found in Borer et al. (2014). Briefly, N, P, and K are added to fertilized plots, each at a rate of 10 g/m^2^/year every year. Micronutrients (μ) are also added to fertilized plots, during the first year of treatment only. The micronutrient mix contains Ca, Mg, S, B, Cu, Fe, Mn, Mo, and Zn, and is added only once in order to avoid toxicity as these micronutrients are largely immobile. Each site has a randomized block design, typically with three blocks (range: 2–6 blocks) and 1–6 replicate plots per block. Plots are 5 × 5 m. A subset of sites also contained a fencing treatment, with fenced unfertilized and fenced fertilized plots. Fences were designed to exclude mid‐to‐large sized (> 50 g) aboveground mammalian herbivores and were typically 230 cm tall with the lower 90 cm surrounded by 1‐cm woven wire mesh, with four strands of tensioned wire strung at equal vertical intervals above the wire mesh, and a 30‐cm outward‐facing flange stapled to the ground to exclude digging animals (e.g., rabbits, voles), but not fully subterranean animals (e.g., gophers, moles). Nutrients were applied inside the fenced fertilized plots in the same way they were applied to unfenced fertilized plots.

### 2.2 Data Collection

Biomass was collected annually at its peak by harvesting all aboveground vegetation in two rectangular strips, typically sized 0.1 × 1 m^2^. We scaled biomass to 1 m^2^ per plot per year for our analyses. Current‐year biomass was separated from previous‐year mass, and we used only current‐year biomass for our study. Biomass was dried to a constant mass at 60°C and weighed to the nearest 0.01 g. Biomass was sampled within the same 1 × 1 m subplot within each plot each year, with the sampling location within the subplot moving every year to avoid effects of previous harvests on current‐year biomass samples. A subset of sites sampled biomass twice per year (to capture total biomass before and after mowing or during both sub‐annual growing seasons) in the same sampling locations. For those sites, we summed biomass values from both sampling dates in each plot to get total biomass per plot per year. We calculated the biomass response ratio as the log response ratio of biomass in control versus nutrient addition plots for each site for each year to use as a proxy for the degree of site nutrient limitation.

Plant community composition was sampled annually in one permanent 1 × 1 m subplot within each plot by visually estimating the aerial percent cover of each species in the subplot. A subset of sites sampled plant community composition twice per year. For those sites, we used the maximum cover value of each species in each plot in each year. We calculated plant species richness for each plot in each year as the number of species recorded per square meter. We also calculated the proportional cover of annual species for each plot in each year as the sum of cover by species with an annual lifespan divided by total cover, and the proportional cover of C_4_ species for each plot in each year as the sum of cover by species with the C_4_ photosynthetic pathway divided by total cover.

To assess light interception by the plant canopy, photosynthetically active radiation (PAR) was measured annually above the plant canopy and at ground level in the same 1 × 1 m subplot used to sample plant community composition. We calculated proportional light interception as 1—(PAR at ground level/PAR above the canopy) for each plot in each year and used this as a proxy for the degree of light limitation.

We used growing season precipitation for all of our analyses. The first and last month of the growing season for each site were identified by the site's coordinator(s) (Table S1), and we calculated total precipitation for each growing season by summing daily precipitation for the length of the growing season. We retrieved daily precipitation data for each site from the Multi‐Source Weighted‐Ensemble Precipitation (MSWEP) product (V280; Beck et al. 2019). For a few sites where MSWEP did not accurately capture precipitation (e.g., due to elevational gradients) we used precipitation data from the closest representative weather station included in the Global Historical Climate Network (GHCN) or from a precipitation gauge at the site, as determined by the site coordinator(s) (Table S1). We required several years of data and a representative range of precipitation values in order to build a reliable relationship between biomass and precipitation at each site. We thus included in our study only sites with a range of growing season precipitation during the study period that spanned at least + and −1 standard deviation from the site‐specific long‐term (~40‐year) average. This resulted in a sample size of 35 sites. We found that restricting our analysis to sites with more years of data or wider ranges of precipitation did not change our main results. For each site, we calculated site aridity as long‐term mean annual precipitation (MAP; average annual precipitation from 1983 [the earliest full calendar year of precipitation data for most of our sites] through 2024) divided by long‐term mean annual PET (from Zomer et al. 2022).

### 2.3 Statistical Analysis

We performed all analyses using R (version 4.3; R Core Team 2024). To test for a change in biomass with treatment and for a change in the sensitivity of biomass to precipitation with treatment across all sites, we performed linear mixed‐effects models with biomass as the response variable. Precipitation, treatment, and the interaction between treatment and precipitation were included as fixed effects. Calendar year, site, and block nested within site were included as random effects. We log_10_ transformed biomass and precipitation data for analyses, to fit model assumptions and also to allow for visualization of nonlinear relationships after back‐transforming model predictions and graphing on linear axes. We also tried fitting a quadratic model and found no evidence that it fit our data better than the linear model (*p* = 0.52), so we present the results of the linear model here. We consider a statistically significant interaction between treatment and precipitation to indicate a significant effect of nutrient addition on biomass sensitivity to precipitation (i.e., a difference in the slope of growing season precipitation vs. biomass in fertilized vs. control plots). We calculated the effect size of nutrient addition on biomass as Cohen's D (Cohen 1988).

To test the influence of extreme precipitation years on the interannual precipitation‐biomass relationship across all sites, we ran another linear mixed‐effects model, this one excluding data from extreme dry and wet years (defined as growing season precipitation less than the 5th or greater than the 95th percentile of each site's site‐specific long‐term record, respectively). To test the effect of nutrient addition on the interannual precipitation‐biomass relationship when water is most limiting, we ran another linear mixed‐effects model, this one using data only from extreme dry years (growing season precipitation less than the 5th percentile of each site's site‐specific long‐term record).

To compare the relationship between precipitation and biomass to the relationship between water deficit and biomass, we ran another linear mixed‐effects model, this one using growing season precipitation minus growing season potential evapotranspiration (PET; from Zomer et al. 2022) as the dependent variable instead of growing season precipitation. Using precipitation−PET yielded the same main result as using precipitation, did not result in higher *R* ^2^ values (conditional *R* ^2^ = 0.54 in both models), and was a worse fit for our data based on AIC (94‐point difference) (Tables S2–S5). We focus on the results of the precipitation model, to facilitate more direct comparisons to past studies of precipitation‐biomass relationships.

To test the influence of herbivory on the interannual precipitation‐biomass relationship, we ran another linear mixed‐effects model, which used data from fenced unfertilized and fenced fertilized plots (*n* = 30 sites that included the fencing treatment), instead of from the unfenced unfertilized and unfenced fertilized plots which were used in all other analyses.

To test for an effect of nutrient addition on rain‐use efficiency (RUE), we calculated RUE for each plot in each year as biomass divided by growing season precipitation. We then ran a linear mixed‐effects model with RUE as the response variable, treatment as a fixed effect, and calendar year, site, and block nested within site as random effects. We calculated the effect size of nutrient addition on RUE as Cohen's D (Cohen 1988).

We used the slope of growing season precipitation versus biomass as our measure of the sensitivity of biomass to precipitation (i.e., the change in biomass per change in precipitation). We used the *R* ^2^ (aka coefficient of determination) of growing season precipitation versus biomass as our measure of the strength of the interannual precipitation‐biomass correlation (i.e., the amount of year‐to‐year variation in biomass that can be explained by the amount of year‐to‐year variation in precipitation). To calculate both the slope (aka sensitivity) and the *R* ^2^ (aka correlation strength) for the fertilized and control plots at each site, we performed two linear models for each site, one using data from fertilized plots and one using data from control plots, with biomass as the response variable and precipitation as the predictor variable. We then tested for a difference in the *R* ^2^ values between fertilized and control plots among all sites using a paired *t*‐test. We calculated the effect size of nutrient addition on biomass sensitivity to precipitation (aka slope) and the strength of the precipitation‐biomass correlation (*R* ^2^) as Cohen's D (Cohen 1988) using the slope and *R* ^2^ values from these models. We also used the slope and *R* ^2^ values from these models as the dependent variables when assessing the effects of additional covariates on biomass sensitivity (slope) and correlation (*R* ^2^) to precipitation (details below).

To assess the context‐dependence of the nutrient addition effect on biomass, biomass sensitivity to precipitation, and the strength of the precipitation‐biomass correlation, we performed three linear mixed effects models with several covariates. The dependent variables in those models were biomass, sensitivity of biomass to precipitation (slope), or the strength of the precipitation‐biomass correlation (*R* ^2^). The fixed effects in the models were treatment, site aridity, light interception, plant species richness, proportional cover by annual species, proportional cover by C_4_ species, biomass response ratio, growing season precipitation (biomass model only), previous growing season precipitation (biomass model only), and number of treatment years (biomass model only) as well as the interaction between treatment and each of the other fixed effects. The random effects in all three models were calendar year, site, and block nested within site. Light interception, species richness, proportion annual species, and proportion C_4_ species were averaged to the site‐treatment scale for the sensitivity and correlation models. Biomass response ratio was averaged to the site scale for the sensitivity and correlation models. Site aridity was at the site scale for all models. We used multi‐model averaging and selection to determine the relative effect of each covariate based on corrected Akaike Information Criteria (AICc). We fit both linear and quadratic lines to the relationships for each dependent‐independent variable combination, and we found that only the relationships between aridity index and sensitivity and between aridity index and correlation strength were fit better by a quadratic versus a linear model (*p* < 0.05) and we thus present the quadratic model for those two relationships and the linear model for the rest of the relationships.

## 3 Results

### 3.1 Impact of Nutrient Addition on Interannual Precipitation‐Biomass Relationships

**FIGURE 3:**
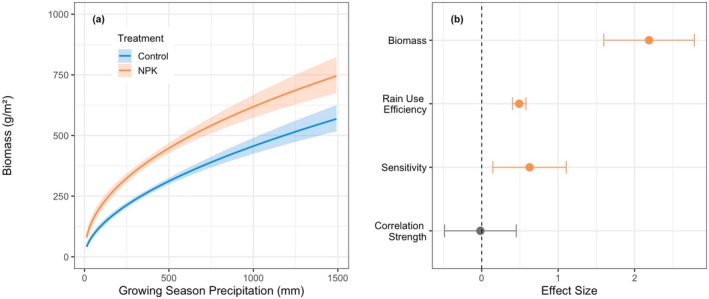
(a) The precipitation‐biomass relationship in control (blue) and nutrient addition (orange) plots, calculated using data from all sites, with standard errors. (b) The size of the nutrient addition effect on biomass, rain‐use efficiency (biomass/precipitation), sensitivity of biomass to precipitation (slope), and the strength of the precipitation‐biomass correlation (*R* ^2^), with confidence intervals. Positive effect sizes (orange) indicate an increase with nutrient addition. Effect sizes were calculated as Cohen's D (Cohen 1988).

Nutrient addition increased plant biomass, by an average of 46% across all sites in our study (384 ± 7.9 g/m^2^ in control plots vs. 563 ± 11.6 g/m^2^ in fertilized plots; *p* < 0.0001; Figure 3; Table S2). This is similar to the 43% increase recently reported in another study that used data from a different subset of NutNet sites and years (Carroll et al. 2025). Nutrient addition also increased the interannual sensitivity of biomass to precipitation (i.e., steeper slope of precipitation vs. biomass in fertilized plots vs. control plots; significant interaction between precipitation and treatment; *p* = 0.017), by about 13% (0.45 ± 0.12 in control plots vs. 0.50 ± 0.11 in fertilized plots; Figure 3; Table S2), indicating a larger increase in biomass per unit increase in precipitation with versus without nutrient addition. Rain‐use efficiency was also higher in fertilized plots than in control plots, by an average of 48% across all sites (0.96 ± 0.03 g/m^2^/mm in control plots vs. 1.4 ± 0.05 g/m^2^/mm in fertilized plots; *p* < 0.0001; Figure 3; Table S6). However, nutrient addition did not change the strength of the interannual precipitation‐biomass correlation (i.e., *R* ^2^; 0.11 ± 0.02 in control plots vs. 0.10 ± 0.03 in fertilized plots; *p* = 0.59; Figure 3; Table S3), indicating no change in the amount of interannual variation in biomass that can be attributed to interannual variation in precipitation with versus without nutrient addition.

### 3.2 Factors Influencing How Nutrient Addition Affects Precipitation‐Biomass Relationships

**FIGURE 4:**
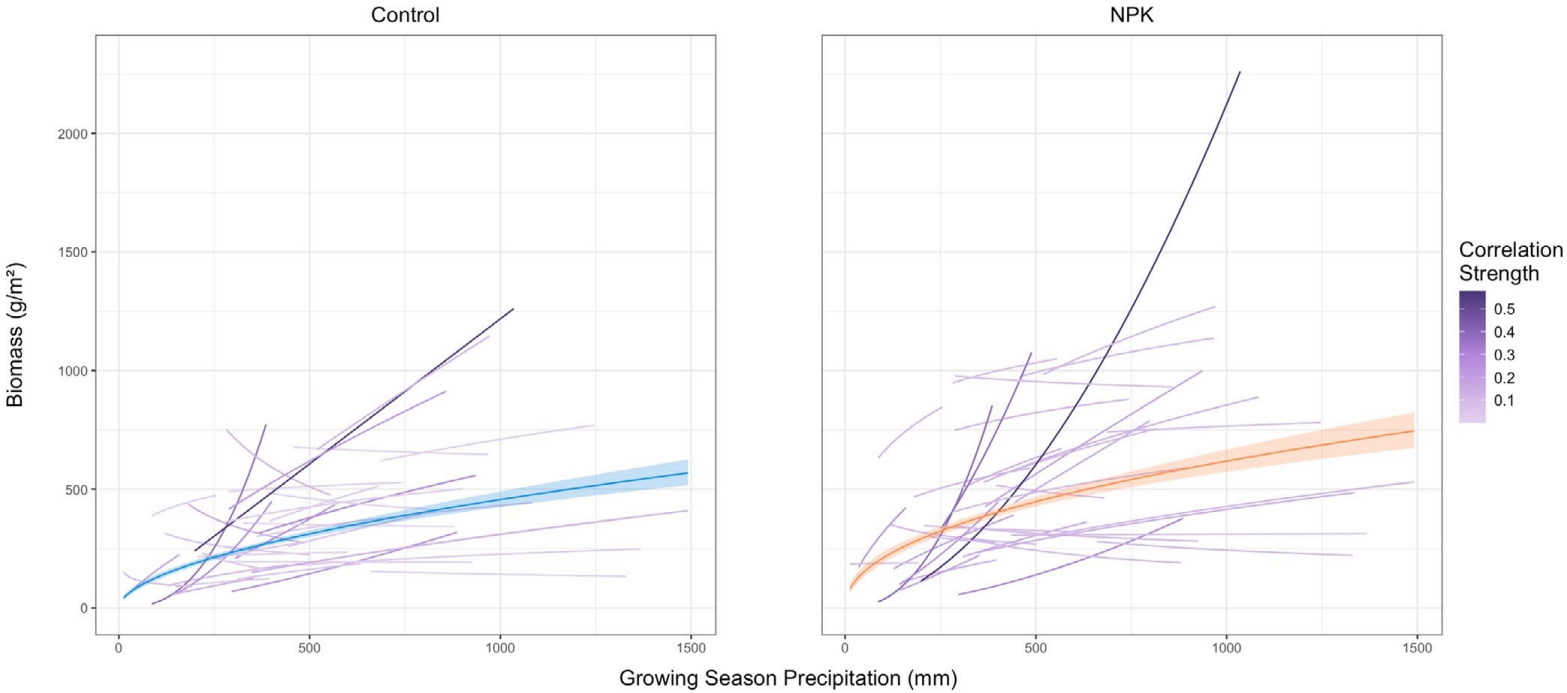
The interannual precipitation‐biomass relationships at all sites, in control and in nutrient addition plots. Each purple line is one site. The shade of purple indicates the strength of the precipitation‐biomass correlation (*R* ^2^) at the site. Overlaid on the site‐level lines are the overall precipitation‐biomass relationship in control (blue) and nutrient addition (orange) plots, calculated using data from all sites, with standard errors (as in Figure 3).

Analyzing only data from non‐extreme precipitation years (growing season precipitation more than the 5th and less than the 95th percentile of the site‐specific long‐term record) yielded the same results across all sites as analyzing data from all years: —increased biomass (*p* < 0.0001) and biomass sensitivity to precipitation (*p* = 0.004), and no change in the strength of the precipitation‐biomass correlation (*p* = 0.55; Figure S1; Tables S7 and S8). But it did change the relationship between precipitation and biomass at some individual sites, where biomass had strong positive (amplified by nutrient addition) or negative responses to extreme wet years (Figures 4 and S6). During extreme dry years, when water is presumably most limiting, we found no evidence for an effect of nutrient addition on biomass (*p* = 0.19) or on the sensitivity of biomass to precipitation (*p* = 0.56), or on the strength of the precipitation‐biomass correlation (*p* = 0.06; Figure S2; Tables S9 and S10) across all sites.

Analyzing fenced unfertilized versus fenced fertilized plots yielded the same results across all sites as analyzing unfenced unfertilized versus unfenced fertilized plots: increased biomass (*p* < 0.0001) and biomass sensitivity to precipitation (*p* = 0.007), and no change in the strength of the precipitation‐biomass correlation (*p* = 0.79; Tables S11 and S12).

As expected, our biomass covariate analysis showed that biomass was higher with nutrient addition, and that biomass was positively correlated with both precipitation and light interception (i.e., higher biomass was associated with larger reductions in light availability at ground level; *p* < 0.0001; Figure S3; Table S13). Biomass also was lower at sites with a higher proportional annual plant species cover (*p* < 0.0001; Figure S3; Table S13). These factors—growing season precipitation, nutrient addition, light interception, and proportional annual cover—were the most important for explaining variation in biomass in our analysis. All four of them were included in all of the best‐fitting biomass models identified by multi‐model averaging and selection (Table S13). The next most‐important covariate in our analysis after those four was the interaction between treatment and proportional C_4_ cover (*p* < 0.0001; Figure S3; Table S13). Nutrient addition had a larger positive effect on biomass when the proportional cover by C_4_ species was low (Figure S3; Table S13).

**FIGURE 5:**
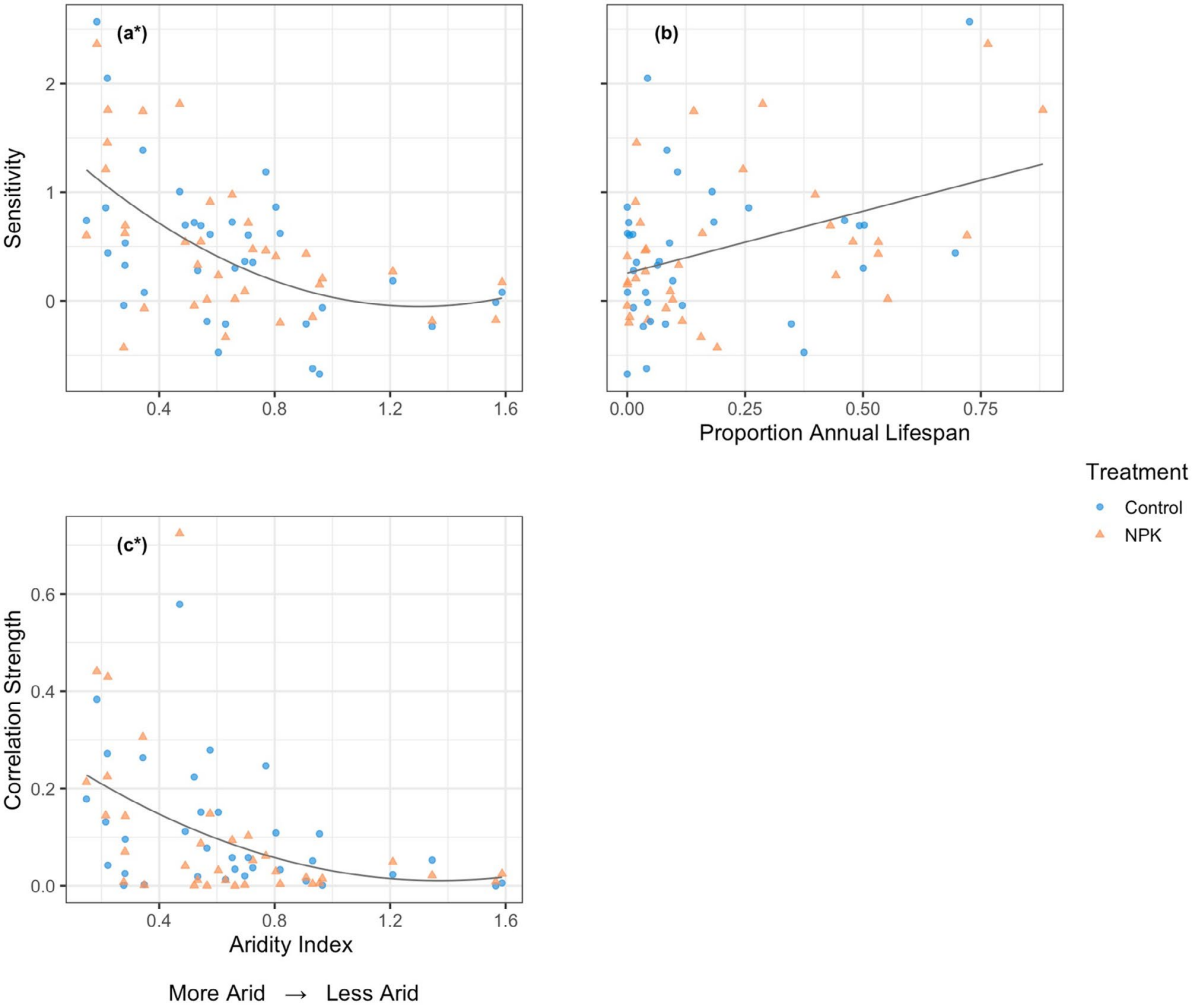
Both sensitivity (a) and correlation (c) of biomass to precipitation increased with site aridity (*p* = 0.0014 and 0.023, respectively), regardless of nutrient addition (treatment by aridity interaction *p* = 0.95 and 0.66, respectively). (b) Biomass sensitivity to precipitation also marginally increased with the proportional cover of annual plant species (*p* = 0.058), regardless of nutrient addition (treatment by proportion annual interaction *p* = 0.74). * = main effect of aridity significant at *p* < 0.05.

As expected, the interannual precipitation‐biomass relationship, and how it changed with nutrient addition, differed among sites (Figures 4 and S6). Our sensitivity and correlation covariate analyses identified site aridity as a key factor driving those differences. Both sensitivity (slope) and correlation (*R* ^2^) of biomass to precipitation increased with site aridity (*p* = 0.0014 and 0.023, respectively), regardless of nutrient addition (treatment by aridity interaction *p* = 0.95 and 0.66, respectively; Figure 5; Tables S14 and S15). Most of our study sites (28 of 35) had a water deficit during the growing season, with PET exceeding precipitation. Site aridity index values (MAP/long‐term mean annual PET) ranged from 0.15 to 1.6, and over half of our study sites (19 of 35) were arid (aridity index < 0.65; Table S1). Sensitivity also marginally increased with the proportional cover of annual plant species (*p* = 0.058), regardless of nutrient addition (treatment by proportion annual interaction *p* = 0.74; Figure 5; Table S14). We found no evidence that any of the other covariates in our analysis helped to explain more of the variation in either the sensitivity or the correlation of biomass to precipitation among sites (Figures S4 and S5; Tables S14 and S15).

## 4 Discussion

### 4.1 Impact of Nutrient Addition on Interannual Precipitation‐Biomass Relationships

Our study of 35 grassland sites that imposed identical fertilization treatments for more than 5 years revealed that nutrient addition increased interannual plant biomass sensitivity to precipitation but did not change the strength of the interannual precipitation‐biomass correlation. That is, nutrient addition increased year‐to‐year variation in biomass but did not make current‐year precipitation a better predictor of current‐year biomass. The interannual precipitation‐biomass relationship varied among sites such that biomass in more arid sites was more sensitive to and more strongly correlated with precipitation, regardless of nutrient addition.

Here we show that nutrient addition increases the slope of the relationship between annual precipitation and annual biomass, extending the recent finding by Fay et al. (2025) that nutrient addition increases the slope of the relationship between long‐term average precipitation and long‐term average biomass. Both studies support the theory that biomass is more strongly limited by nutrients at higher levels of precipitation (e.g., Hooper and Johnson 1999; Huxman et al. 2004), by showing that nutrient addition has a larger positive effect on biomass when water availability is higher, on both interannual (this study) and long‐term average (Fay et al. 2025) timescales.

The Huxman‐Smith model (Huxman et al. 2004), which showed higher biomass sensitivity to precipitation at more arid sites, as well as weaker correlations between precipitation and biomass within sites versus among sites, has become foundational to our understanding of precipitation‐biomass relationships (Maurer et al. 2020; Knapp et al. 2015, 2024; Sala et al. 2012). Here, we show that nutrient addition does not change that pattern—interannual biomass sensitivity to precipitation was higher at more arid sites regardless of nutrient addition. We further expand upon the Huxman‐Smith model by showing that, in addition to sensitivity, the correlation between biomass and precipitation is also higher at more arid sites. Further, our finding that nutrient addition increased rain‐use efficiency of biomass provides empirical confirmation of a key prediction of the resource use hypothesis that Huxman et al. (2004) used to explain the increased sensitivity of biomass to precipitation with increasing aridity. They predicted that removing limitations by factors other than water (e.g., nutrients) would increase rain‐use efficiency, which we confirmed in this study.

The average 13% increase in biomass sensitivity to interannual precipitation fluctuations that we found across all sites with nutrient addition could have meaningful ecological consequences. This increased sensitivity could amplify the effects of even small increases in year‐to‐year precipitation variability on biomass and substantially increase interannual fluctuations in ecosystem functioning, including forage production, habitat and fuel availability, and cycling and storage of carbon.

### 4.2 Impact of Extreme Precipitation Years

Our main results were the same with or without extreme precipitation years in our analyses, suggesting that the effect of nutrient addition on the precipitation‐biomass relationship is generally robust to very wet and dry years across many sites. However, at some individual sites, biomass did show a strong positive or negative response to extreme wet years that diverged from the non‐extreme precipitation‐biomass relationship. Biomass was low and not affected by nutrient addition during extreme wet years at some sites, likely because excessive precipitation can over‐saturate soil and cause waterlogging, decreasing aeration, increasing denitrification, and lowering photosynthetic activity (Parent et al. 2008; Zhang et al. 2025), resulting in low biomass (Knapp et al. 2017). Other sites showed the opposite pattern, with large biomass increases in wet years, especially in fertilized plots, a pattern reported by Collins et al. (2025) and by Wang et al. (2017) in semi‐arid and arid grasslands. Though site aridity helped explain patterns of biomass sensitivity to precipitation among all sites and years, it did not explain different responses to extreme wet years among sites. It is possible that intra‐annual precipitation patterns and/or soil texture could help explain these different responses, as waterlogging might be more likely to occur if precipitation falls in a few large versus several small events (e.g., Brown and Collins 2024) and/or if the soil at the site has a high water holding capacity (e.g., high clay and low sand content).

During extreme dry years, when water was presumably most limiting, we found that there was no effect of nutrient addition on biomass or biomass sensitivity to precipitation when comparing across sites. This is consistent with the idea that plants are limited only by the most‐limiting resource (aka Liebig's Law of the Minimum Hypothesis; Farrior et al. 2013; van der Ploeg et al. 1999), but was only evident under extreme dry conditions. Similar to our study, Bharath et al. (2020) found that nutrient addition did not affect biomass during dry years when comparing among many grassland sites, though nutrient addition did exacerbate biomass declines at a subset of sites in that study and in ours. Our findings also offer a possible explanation as to why nutrient addition does not consistently increase biomass in all grasslands in all years (Carroll et al. 2025), as some sites do not respond to nutrient addition during extreme dry or wet years.

### 4.3 Other Factors Influencing How Nutrient Addition Affects Precipitation‐Biomass Relationships

We found that site aridity and plant species lifespan help explain differences in the precipitation‐biomass relationship among sites. Consistent with previous studies, the interannual precipitation‐biomass relationship varied among our study sites (Huxman et al. 2004; Knapp et al. 2015; Maurer et al. 2020; Sala et al. 2012), as did the effect of nutrient addition on the interannual precipitation‐biomass relationship (Collins et al. 2025; Hooper and Johnson 1999; Morgan et al. 2016; Yahdjian et al. 2011). At more arid sites, both biomass sensitivity to precipitation and the strength of the precipitation‐biomass correlation were higher, regardless of nutrient addition. Sensitivity was also marginally higher at sites where a larger proportion of the vegetation cover was by plants with an annual lifespan (consistent with Cleland et al. 2013 and Wilcox et al. 2021), also regardless of nutrient addition. Annual plants tend to have “faster” growth traits associated with higher sensitivity to precipitation, while the ability to use carbohydrates stored from previous years can help buffer perennial plants against interannual precipitation variability (Wilcox et al. 2021). The other covariates in our analysis didn't help explain more of the variation in biomass sensitivity or correlation to precipitation. Similarly, our main results were the same when comparing control and nutrient addition plots that were fenced as when comparing plots that were not fenced (consistent with Irisarri et al. 2016).

Our study illuminates patterns of biomass sensitivity to interannual variation in precipitation, but even after removing nutrient limitation and considering numerous additional factors, there was still unexplained variation in the overall interannual precipitation‐biomass relationship. The lack of a strong correlation between current‐year precipitation and current‐year biomass in grasslands thus remains a paradox (Wang and Collins 2024). Future studies could investigate the possibility of complex dependencies among covariates that were beyond the scope of this study. For example, the effect of previous year precipitation might depend on site aridity (He et al. 2025). Intra‐annual precipitation patterns (Feldman et al. 2024; Knapp et al. 2002) and seasonal timing of precipitation (Densmore‐McCulloch et al. 2016; Peng et al. 2013) might also affect biomass and can interact with both precipitation amount (Felton et al. 2020) and site aridity (Knapp et al. 2008). We didn't measure soil moisture content but it is possible that is a stronger predictor of biomass than precipitation. Similarly, we didn't have enough soil texture data to include in our analyses, but it is possible this could affect the amount of precipitation available for plant uptake, with varying effects based on site aridity (Noy‐Meir 1973).

## 5 Conclusion

With both nutrient inputs and precipitation variability increasing globally (Douville et al. 2021; Giorgi et al. 2019; Pfahl et al. 2017; Rockström et al. 2009; Zhang et al. 2021), it is important to understand how nutrient addition affects the interannual precipitation‐biomass relationship. Our study supported our hypotheses that nutrient addition would increase biomass, rain‐use efficiency, and biomass sensitivity to precipitation, but did not support our hypothesis that nutrient addition would also increase the strength of the precipitation‐biomass correlation, which did not change. Our study also supported our hypothesis that the interannual precipitation‐biomass relationship would vary among sites. Specifically, we found that biomass was more sensitive to precipitation and more strongly correlated with precipitation in more arid sites, regardless of nutrient addition, and that none of the other co‐variates in our analysis helped explain the remaining variation in biomass sensitivity or correlation to precipitation.

By showing that nutrient addition amplifies biomass sensitivity to interannual precipitation variability across a wide range of biotic and abiotic conditions, our results indicate that grassland biomass will likely become more variable from year to year as nutrient enrichment increases biomass sensitivity to precipitation and precipitation becomes more variable.

## Author Contributions

**Ingrid J. Slette:** conceptualization, investigation, writing – original draft, methodology, visualization, writing – review and editing, formal analysis, project administration, data curation. **Dana Blumenthal:** writing – review and editing, project administration. **Philip A. Fay:** writing – review and editing, project administration. **Sylvia Haider:** writing – review and editing, project administration. **Carlos Alberto Arnillas:** writing – review and editing, project administration. **Jonathan D. Bakker:** writing – review and editing, project administration. **Jane A. Catford:** writing – review and editing, project administration. **Scott L. Collins:** writing – review and editing, project administration. **Nico Eisenhauer:** writing – review and editing, project administration. **Magda Garbowski:** writing – review and editing, project administration. **Anita C. Risch:** writing – review and editing, project administration. **Eric W. Seabloom:** conceptualization, writing – review and editing, project administration, supervision, funding acquisition. **Michelle Tedder:** writing – review and editing, project administration. **Kimberly J. Komatsu:** writing – review and editing, project administration. **Andrew S. MacDougall:** writing – review and editing, project administration. **Elizabeth T. Borer:** writing – review and editing, project administration, conceptualization, funding acquisition, supervision. **Kevin P. Kirkman:** writing – review and editing, project administration. **Johannes M. H. Knops:** writing – review and editing, project administration. **W. Stanley Harpole:** writing – review and editing, project administration. **Christiane Roscher:** writing – review and editing, project administration. **Jason P. Martina:** writing – review and editing, project administration. **Sally A. Power:** writing – review and editing, project administration. **Melinda D. Smith:** writing – review and editing, project administration. **Andrew D. B. Leakey:** writing – review and editing, project administration.

## Funding

This work was supported by National Science Foundation, NSF‐DEB‐1042132, NSF‐DEB‐2425352, NSF‐DEB‐1831944.

## Conflicts of Interest

The authors declare no conflicts of interest.

## Supporting information


**Figure S1:** The precipitation‐biomass relationship during non‐extreme precipitation years (precipitation above the 5th percentile and below the 95th percentile of the long‐term site‐specific record), with standard errors. Trend lines indicate a significant positive correlation between precipitation and biomass in both control (blue) and nutrient addition plots (orange). Fertilization increased biomass and biomass sensitivity to precipitation, and did not change the strength of the precipitation‐biomass relationship.
**Figure S2:** The precipitation‐biomass relationship during extreme dry years (precipitation below the 5th percentile of the long‐term site‐specific record), with standard errors. Biomass was positively correlated with precipitation (grey trend line). However, there was no effect of fertilization on biomass, biomass sensitivity to precipitation, or the strength of the precipitation‐biomass relationship during extreme dry years.
**Figure S3:** Correlations between biomass and each factor in our covariate analysis. Grey trend lines indicate that biomass was significantly (p < 0.05) correlated with aridity index, light interception, and proportion annual lifespan, and that the correlation between biomass and each of these factors did not vary by treatment (no interaction between treatment and any of those factors). The orange (nutrient addition) and blue (control) lines indicate that the correlation between biomass and proportion C4 varied by treatment (significant interaction between proportion C4 and treatment).
**Figure S4:** Correlations between biomass sensitivity to precipitation and each factor in our covariate analysis. Trend lines indicate a significant relationship (p = 0.002) between biomass and aridity index, and a marginally significant relationship (p = 0.058) between biomass and the proportion annual lifespan. Biomass sensitivity to precipitation was higher at more arid sites (lower aridity index = more arid) and at sites with a higher proportional cover by annual plant species, regardless of fertilization (no interaction between treatment and aridity or between treatment and proportion annual lifespan).
**Figure S5:** Correlations between the strength of the precipitation‐biomass correlation (R2) and each factor in our covariate analysis. The trend line indicates a significant relationship (p < 0.05) between correlation strength and site aridity (lower aridity index = more arid). Regardless of fertilization, correlation strength was higher at more arid sites (no interaction between treatment and aridity).
**Figure S6:** Interannual precipitation‐biomass relationships at each study site. Trend lines indicate a significant relationship between biomass and precipitation in control (blue) and/or nutrient addition (orange) plots at a site, based on separate linear models of control and nutrient addition data at each site. Sites are ordered from lowest to highest site aridity index (lower values = more arid), moving from left to right and top to bottom.
**Table S1:** Site information. MAP = mean annual precipitation (mm), MAT = mean annual temperature (°C). MSWEP = Multi‐Source Weighted‐Ensemble Precipitation product (V280; Beck et al. 2019). GHCN = Global Historical Climate Network.
**Table S2:** Results of main linear mixed‐effects model assessing precipitation and fertilization effect on biomass and biomass sensitivity to precipitation. Model conditional, marginal R2 = 0.54, 0.19. Model AIC = 997.
**Table S3:** Results of paired *t*‐test of differences in the strength of the precipitation‐biomass correlation (R2) in control versus fertilized plots among sites.
**Table S4:** Results of linear mixed‐effects model assessing precipitation‐potential evapotranspiration and fertilization effect on biomass and biomass sensitivity to precipitation‐potential evapotranspiration among sites. PET = potential evapotranspiration. Model conditional, marginal R2 = 0.54, 0.12. Model AIC = 1091.
**Table S5:** Results of paired *t*‐test of differences in the strength of the correlation (R2) between precipitation‐potential evapotranspiration in control versus fertilized plots among sites.
**Table S6:** Results of linear mixed‐effects model assessing fertilization effect on rain‐use efficiency.
**Table S7:** Results of linear mixed‐effects model assessing precipitation and fertilization effects on biomass and biomass sensitivity to precipitation during non‐extreme precipitation years.
**Table S8:** Results of paired *t*‐test of differences in the precipitation‐biomass correlation (R2) in control versus fertilized plots among sites, during non‐extreme precipitation years.
**Table S9:** Results of linear mixed‐effects model, assessing precipitation and fertilization effect on biomass and biomass sensitivity to precipitation during extreme dry years.
**Table S10:** Results of paired *t*‐test of differences in the precipitation‐biomass correlation (R2) in control versus fertilized plots among sites, during extreme dry years.
**Table S11:** Results of linear mixed‐effects model assessing precipitation and fertilization effect on biomass and biomass sensitivity to precipitation in fenced plots.
**Table S12:** Results of paired *t*‐test of differences in the strength of the precipitation‐biomass correlation (R2) in fenced plots.
**Table S13:** Results of biomass covariate analysis. Conditional model‐averaged predictors of biomass among all study sites. Total candidate models evaluated: 1174. Predictors allowed per model: ≤ 6. Models included in averaging: ΔAICc < 50. Relative importance = summed Akaike weights across the model set.
**Table S14:** Results of sensitivity covariate analysis. Conditional model‐averaged predictors of biomass sensitivity to precipitation among all study sites. Total candidate models evaluated: 358. Predictors allowed per model: ≤ 6. Models included in averaging: ΔAICc < 10. Relative importance = summed Akaike weights across the model set.
**Table S15:** Results of correlation strength covariate analysis. Conditional model‐averaged predictors of the strength of the precipitation‐biomass correlation among all study sites. Total candidate models evaluated: 358. Predictors allowed per model: ≤ 6. Models included in averaging: ΔAICc < 10. Relative importance = summed Akaike weights across the model set.
**Table S16:** Author contributions to manuscript.

## Data Availability

The data used in this study are available in the Environmental Data Initiative (EDI) repository at: https://doi.org/10.6073/pasta/2edbdb37cdd1256832e5f6b751d6216e.
