# Supplementary material for: Nutrient Enrichment Increases Grassland Biomass Sensitivity to Interannual Precipitation Variation
Source: Glob Chang Biol. 2026 Aug 17;32(8):e71053. doi: 10.1111/gcb.71053 (PMC13481996; doi:10.1111/gcb.71053)
Supplement: Supplementary file 1 [file GCB-32-e71053-s001.pdf]

Supplemental material:

Slette et al. 2026 “Nutrient enrichment increases grassland biomass sensitivity to interannual precipitation variation” Global Change Biology

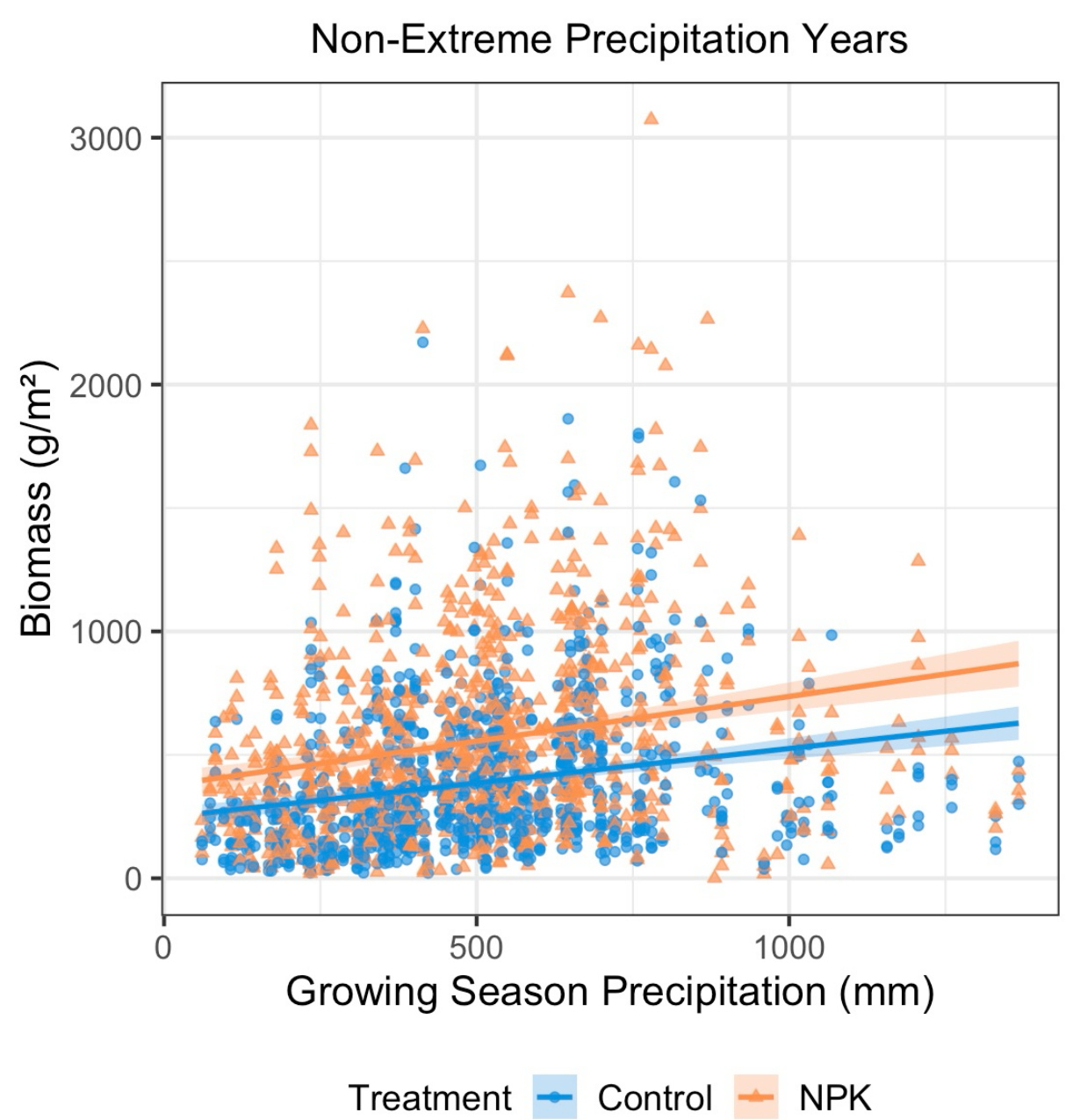

Figure S1: The precipitation-biomass relationship during non-extreme precipitation years (precipitation above the 5th percentile and below the 95th percentile of the long-term site-specific record), with standard errors. Trend lines indicate a significant positive correlation between precipitation and biomass in both control (blue) and nutrient addition plots (orange). Fertilization increased biomass and biomass sensitivity to precipitation, and did not change the strength of the precipitation-biomass relationship.

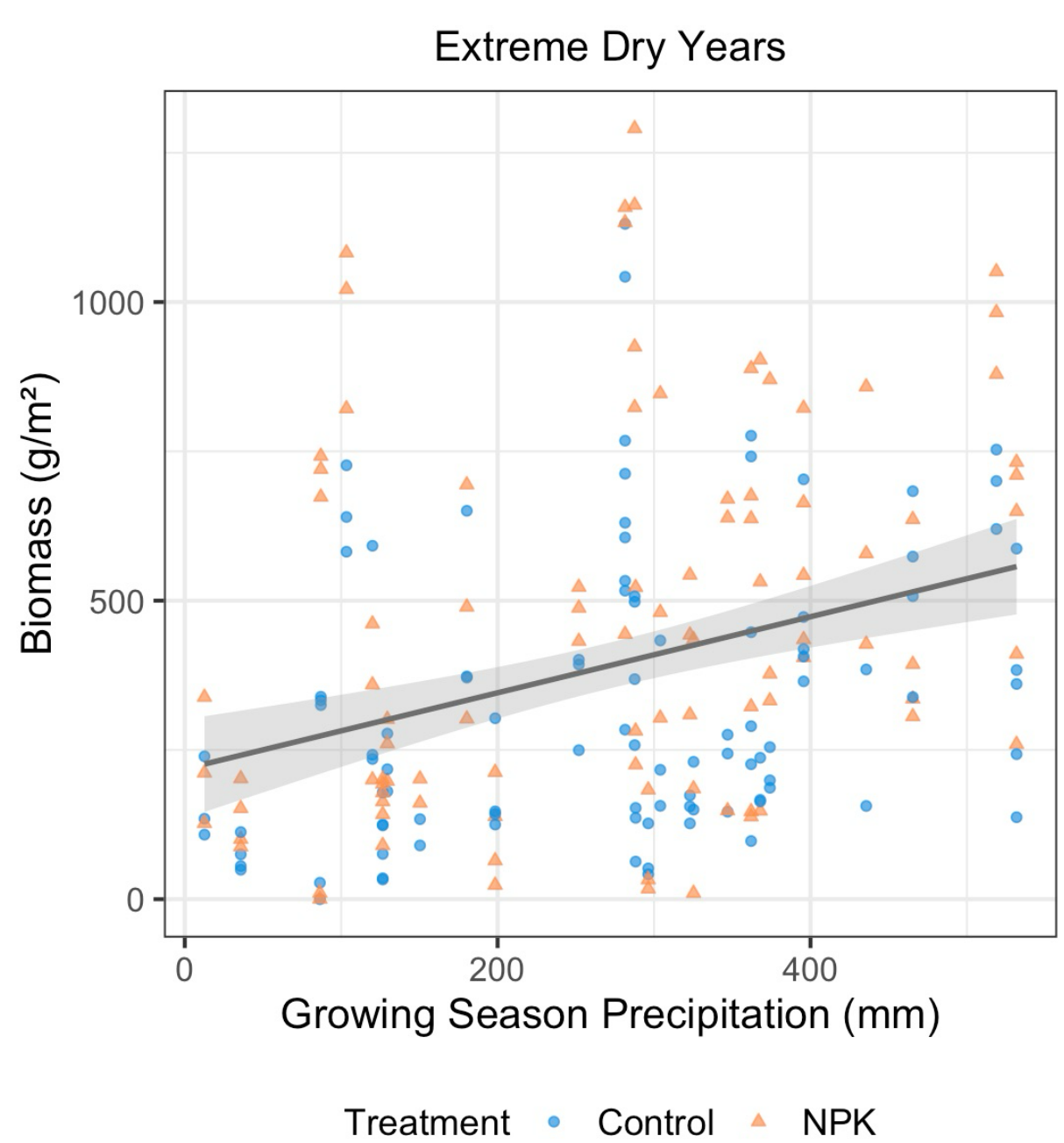

Figure S2: The precipitation-biomass relationship during extreme dry years (precipitation below the 5th percentile of the long-term site-specific record), with standard errors. Biomass was positively correlated with precipitation (grey trend line). However, there was no effect of fertilization on biomass, biomass sensitivity to precipitation, or the strength of the precipitation-biomass relationship during extreme dry years.

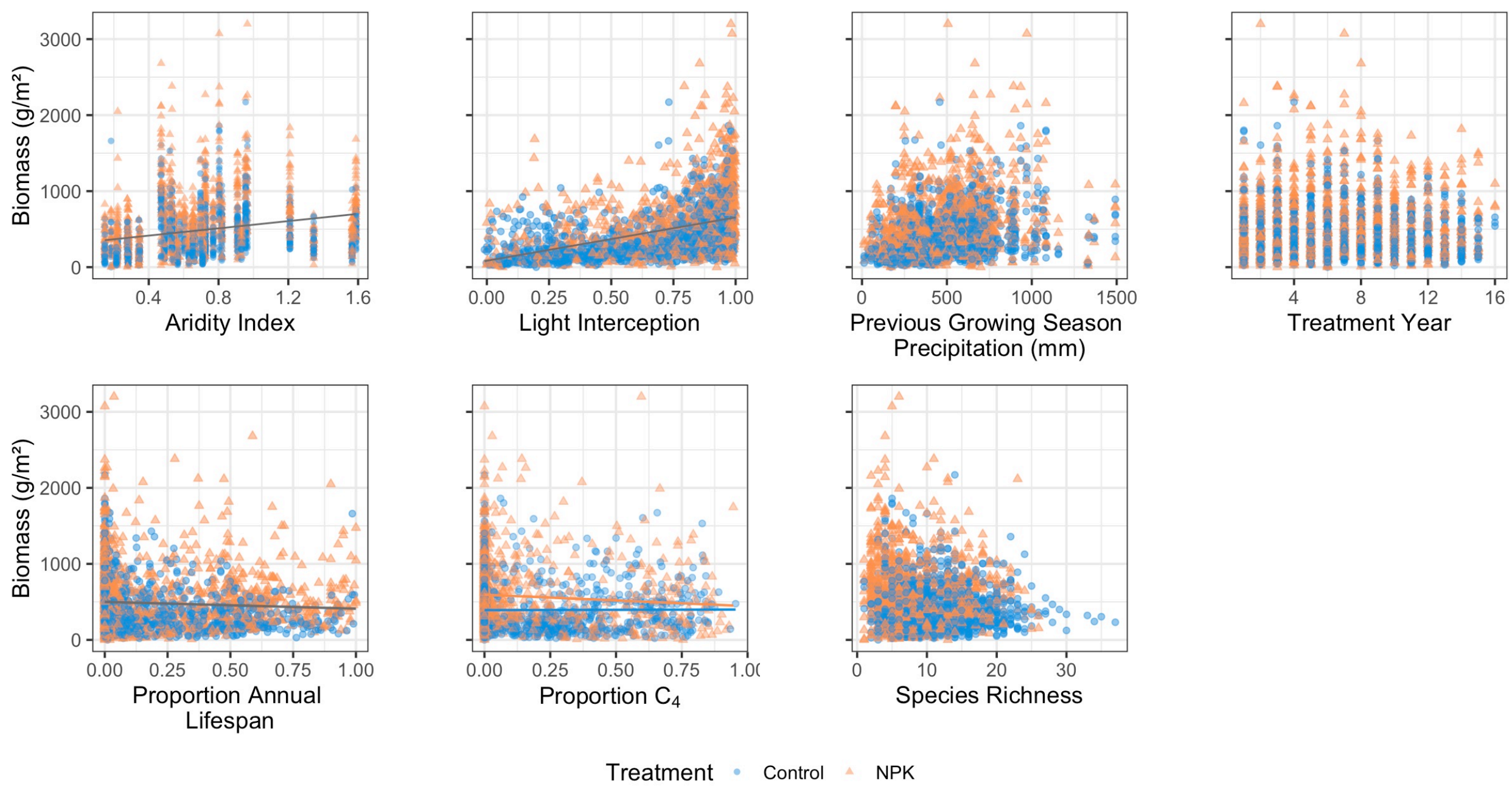

Figure S3: Correlations between biomass and each factor in our covariate analysis. Grey trend lines indicate that biomass was significantly ($p<0.05$) correlated with aridity index, light interception, and proportion annual lifespan, and that the correlation between biomass and each of these factors did not vary by treatment (no interaction between treatment and any of those factors). The orange (nutrient addition) and blue (control) lines indicate that the correlation between biomass and proportion C_4 varied by treatment (significant interaction between proportion C_4 and treatment).

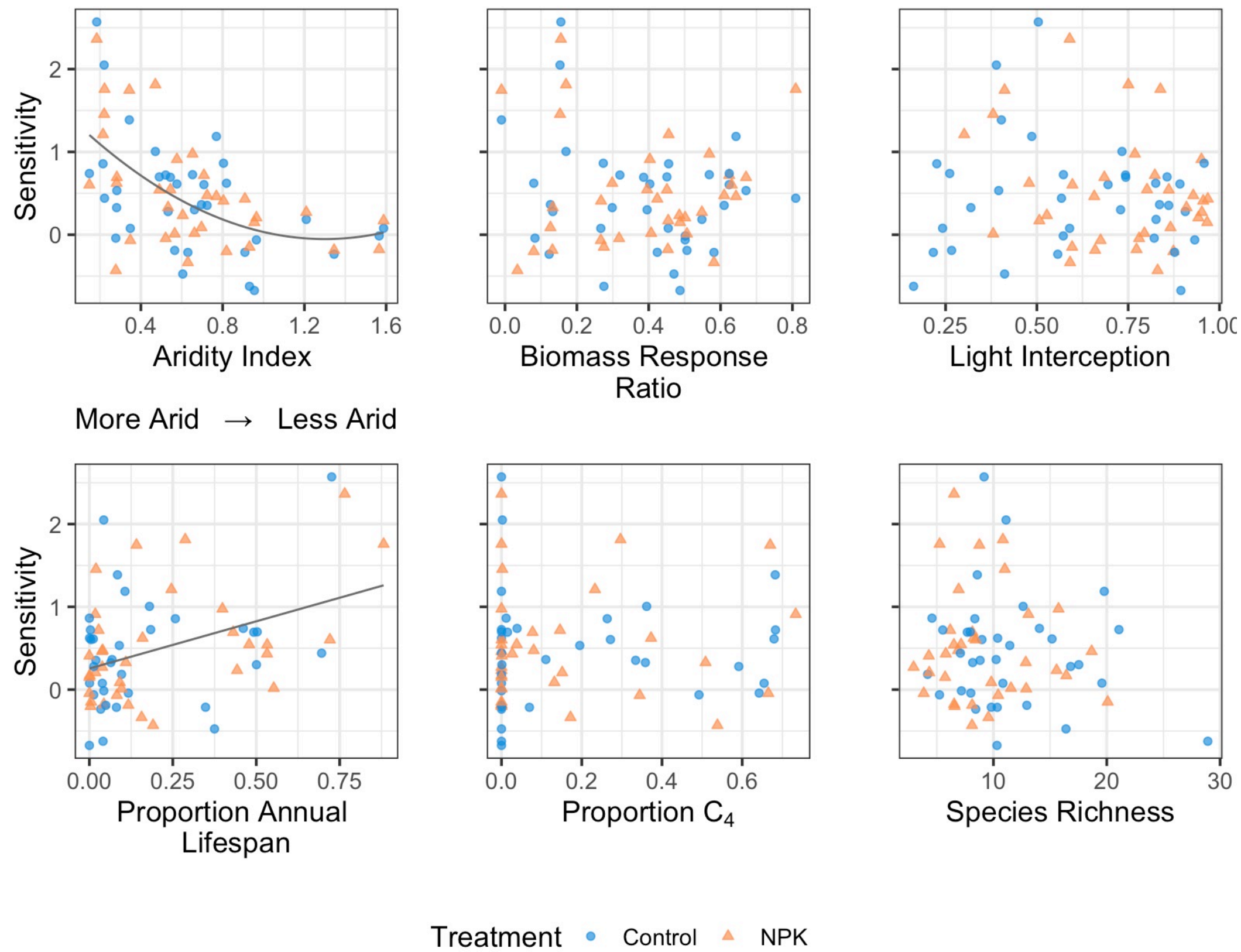

Figure S4: Correlations between biomass sensitivity to precipitation and each factor in our covariate analysis. Trend lines indicate a significant relationship (p=0.002) between biomass and aridity index, and a marginally significant relationship (p=0.058) between biomass and the proportion annual lifespan. Biomass sensitivity to precipitation was higher at more arid sites (lower aridity index = more arid) and at sites with a higher proportional cover by annual plant species, regardless of fertilization (no interaction between treatment and aridity or between treatment and proportion annual lifespan).

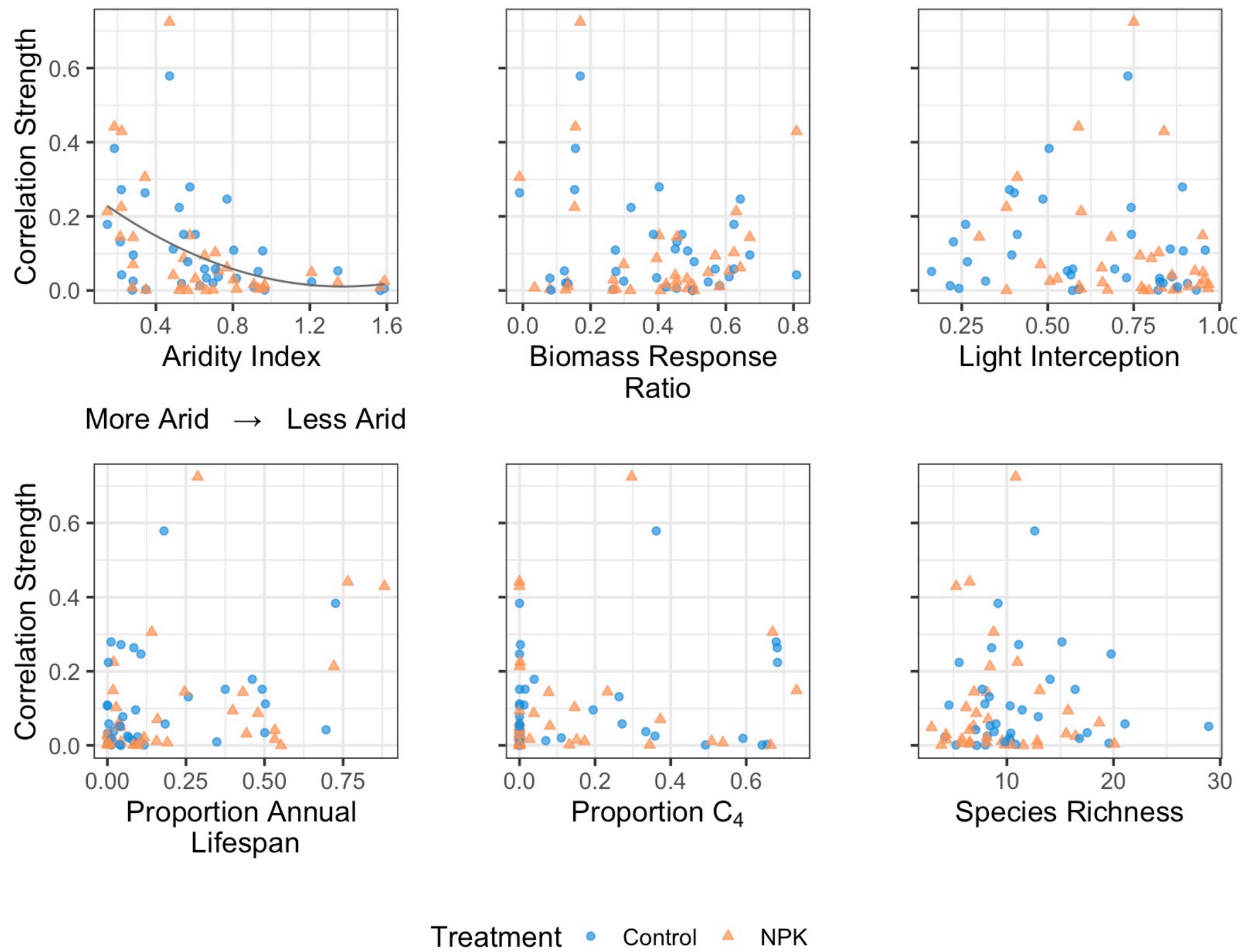

Figure S5: Correlations between the strength of the precipitation-biomass correlation (R^2) and each factor in our covariate analysis. The trend line indicates a significant relationship ($p<0.05$) between correlation strength and site aridity (lower aridity index = more arid). Regardless of fertilization, correlation strength was higher at more arid sites (no interaction between treatment and aridity).

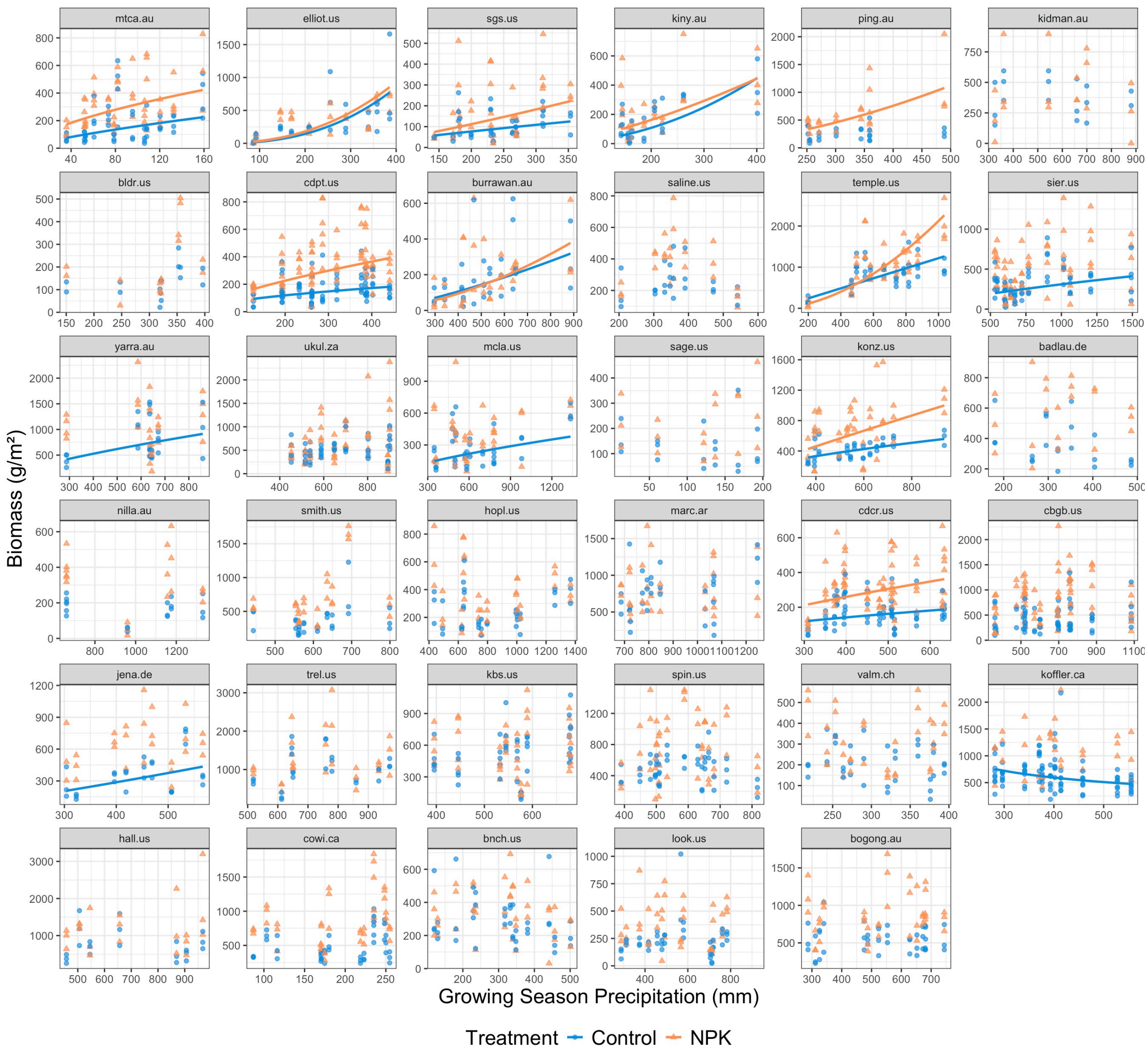

Figure S6: Interannual precipitation-biomass relationships at each study site. Trend lines indicate a significant relationship between biomass and precipitation in control (blue) and/or nutrient addition (orange) plots at a site, based on separate linear models of control and nutrient addition data at each site. Sites are ordered from lowest to highest site aridity index (lower values = more arid), moving from left to right and top to bottom.

Table S1: Site information. MAP = mean annual precipitation (mm), MAT = mean annual temperature (°C). MSWEP = Multi-Source Weighted-Ensemble Precipitation product (V280; Beck et al. 2019). GHCN = Global Historical Climate Network.

Site Code	Site Name	Latitude	Longitude	MAP	MAT	Aridity Index	Growing Season Start	Growing Season End	Precip. Source	Ecosystem Type	Years of Data
badlau.de	*Bad Lauchstaedt	51.392	11.878	523	9.33	0.6	Apr	Oct	MSWEP	Old Field	7
bldr.us	*Boulder South Campus	39.972	-105.234	487	9.9	0.28	Mar	Aug	Local	Shortgrass prairie	8
bnch.us	Bunchgrass (Andrews LTER)	44.277	-121.968	1618	6.77	1.35	Apr	Aug	GHCN	Grassland	14
bogong.au	Bogong	-36.874	147.254	1678	5.98	1.59	Oct	Jan	MSWEP	Alpine grassland	14
burrawan.au	Burrawan	-27.736	151.139	643	18.22	0.34	Oct	May	MSWEP	Pasture	11
cbgb.us	Chichaqua Bottoms	41.785	-93.385	871	9.26	0.72	May	Oct	MSWEP	Tallgrass prairie	14
cdcr.us	Cedar Creek LTER	45.425	-93.212	740	6.34	0.71	Apr	Aug	MSWEP	Tallgrass prairie	15
cdpt.us	Cedar Point Biological Station	41.206	-101.643	456	9.64	0.28	Apr	Aug	MSWEP	Shortgrass prairie	14
cowi.ca	Cowichan	48.809	-123.63	762	10.45	1.21	Apr	Jul	MSWEP	Old Field	14
elliot.us	Elliott Chaparral	32.875	-117.052	344	17.71	0.18	Nov	Apr	GHCN	Annual grassland	12
hall.us	*Hall's Prairie	36.872	-86.702	1289	13.83	0.97	Apr	Sept	MSWEP	Tallgrass prairie	7
hopl.us	Hopland REC	39.013	-123.06	1065	13.24	0.66	Nov	Apr	MSWEP	Annual grassland	13
jena.de	*JeNut	50.938	11.528	654	8.57	0.77	Mar	Oct	MSWEP	Grassland	9
kbs.us	Kellogg Biological Station LTER	42.409	-85.391	903	8.8	0.82	Apr	Sept	MSWEP	Old field	9
kidman.au	*Kidman Springs	-16.108	130.953	724	27.26	0.28	Nov	Apr	MSWEP	Savanna	6
kiny.au	Kinypanial	-36.2	143.75	408	15.59	0.22	May	Oct	MSWEP	Semiarid grassland	10
koffler.ca	*Koffler Scientific Reserve at Joker's Hill	44.024	-79.536	853	6.28	0.96	Apr	Aug	MSWEP	Pasture	11
konz.us	*Konza LTER	39.071	-96.583	889	12.08	0.58	May	Sept	MSWEP	Tallgrass prairie	14

look.us	Lookout (Andrews LTER)	44.205	-122.128	1877	6.9	1.57	Mar	Aug	MSWEP	Montane grassland	14
marc.ar	Mar Chiquita	-37.715	-57.425	907	14.32	0.7	Jan	Dec	MSWEP	Grassland	11
mcla.us	Mclaughlin UCNRS	38.864	-122.406	936	13.97	0.54	Nov	Apr	MSWEP	Annual grassland	13
mtca.au	Mt. Caroline	-31.782	117.611	324	17.75	0.15	Aug	Oct	GHCN	Savanna	13
nilla.au	*Nillahcootie	-36.896	146.007	947	13.78	0.63	Feb	Dec	MSWEP	Old field	6
ping.au	*Pingelly Paddock	-32.496	116.973	456	16.28	0.22	Apr	Oct	MSWEP	Old field	8
sage.us	Sagehen Creek UCNRS	39.43	-120.24	831	5.83	0.57	Apr	Jul	GHCN	Montane grassland	6
saline.us	Saline Experimental Range	39.05	-99.1	608	12.1	0.35	May	Sept	MSWEP	Mixedgrass prairie	8
sgs.us	Shortgrass Steppe LTER	40.817	-104.767	369	8.95	0.21	Apr	Aug	MSWEP	Shortgrass prairie	14
sier.us	Sierra Foothills REC	39.236	-121.284	936	16.31	0.49	Nov	Apr	MSWEP	Annual grassland	13
smith.us	Smith Prairie	48.207	-122.625	605	10.18	0.65	Oct	Jun	MSWEP	Mesic grassland	10
spin.us	*Spindletop	38.132	-84.503	1152	12.48	0.91	Jan	May	MSWEP	Pasture	16
temple.us	Temple	31.044	-97.349	877	19.4	0.47	Mar	Oct	MSWEP	Tallgrass prairie	11
trel.us	Trelease	40.075	-88.829	992	11.07	0.8	Jan	Sept	MSWEP	Tallgrass prairie	9
ukul.za	*Ukulinga	-29.67	30.401	832	17.65	0.53	Sept	Apr	Local	Mesic grassland	12
valm.ch	Val Mustair	46.631	10.372	681	0.13	0.93	Jun	Aug	MSWEP	Alpine grassland	12
yarra.au	*Yarramundi	-33.614	150.738	844	17.32	0.52	Sept	Mar	MSWEP	Mesic grassland	7

Biomass is sampled twice per year at JeNet (before mowing in June and at the end of the growing season), and at Temple (due to bi-modal weather pattern)

* = site is managed (e.g., by periodic prescribed fire or mowing)

Table S2: Results of main linear mixed-effects model assessing precipitation and fertilization effect on biomass and biomass sensitivity to precipitation. Model conditional, marginal R^2= 0.54, 0.19. Model AIC = 997.

Fixed Effects	Estimate	Standard Error	Degrees of Freedom	t value	P value
Intercept	0.92	0.12	1028.70	7.51	<0.0001
Precipitation	0.58	0.04	2333.80	13.29	<0.0001
Fertilization	0.42	0.10	2580.60	4.16	<0.0001
Precipitation × Fertilization	−0.091	0.04	2580.70	−2.38	0.017

Random Effects	Variance	Standard Deviation
Site	0.05	0.23
Block (within site)	0.001	0.04
Year	0.004	0.06
Residual	0.08	0.28

Table S3: Results of paired t-test of differences in the strength of the precipitation-biomass correlation (R^2) in control vs. fertilized plots among sites.

Metric	Mean difference (Fertilized − Control)	95% Confidence Interval	t value	Degrees of Freedom	P value
R^2	0.009	−0.025 to 0.043	0.545	34	0.589

Table S4: Results of linear mixed-effects model assessing precipitation-potential evapotranspiration and fertilization effect on biomass and biomass sensitivity to precipitation-potential evapotranspiration among sites. PET = potential evapotranspiration. Model conditional, marginal R^2= 0.54, 0.12. Model AIC = 1091.

Fixed Effects	Estimate	Standard Error	Degrees of Freedom	t value	P value
Intercept	2.49	0.05	43.3	51.38	<0.001
Precipitation - PET	0.000328	0.00004	2569.0	8.99	<0.001
Fertilization	0.19	0.01	2581.0	15.93	<0.001
Precipitation - PET × Fertilization	0.00005	0.00003	2580.0	1.46	0.045

Random Effects	Variance	Standard Deviation
Site	0.73	0.26
Block (within site)	0.002	0.04
Year	0.004	0.06
Residual	0.08	0.28

Table S5: Results of paired t-test of differences in the strength of the correlation (R^2) between precipitation-potential evapotranspiration in control vs. fertilized plots among sites.

Metric	Mean difference (Fertilized − Control)	95% Confidence Interval	t value	Degrees of Freedom	P value
R^2	0.013	-0.024 to 0.050	0.701	35	0.488

Table S6: Results of linear mixed-effects model assessing fertilization effect on rain-use efficiency.

Fixed Effects	Estimate	Standard Error	Degrees of Freedom	t value	P value
Intercept	0.962	0.153	43.3	6.29	<0.001
Fertilization	0.509	0.043	2258	11.78	<0.001

Random Effects	Variance	Standard Deviation
Site	0.682	0.826
Block (within site)	0.000	0.000
Year	0.044	0.211
Residual	1.062	1.031

Table S7: Results of linear mixed-effects model assessing precipitation and fertilization effects on biomass and biomass sensitivity to precipitation during non-extreme precipitation years.

Fixed Effects	Estimate	Standard Error	Degrees of Freedom	t value	P value
Intercept	1.385	0.169	956.7	8.21	<0.0001
Precipitation	0.405	0.062	1363.5	6.53	<0.0001
Fertilization	0.524	0.122	1784.8	4.30	<0.0001
Precipitation × Fertilization	-0.131	0.046	1784.8	-2.86	0.0043

Random Effects	Variance	Standard Deviation
Site	0.053	0.23
Block (within site)	0.001	0.03
Year	0.005	0.07
Residual	0.058	0.24

Table S8: Results of paired t-test of differences in the precipitation-biomass correlation (R^2) in control vs. fertilized plots among sites, during non-extreme precipitation years.

Metric	Mean difference (Fertilized – Control)	95% Confidence Interval	t value	Degrees of Freedom	P value
R^2	-0.013	-0.058 to 0.031	-0.609	34	0.547

Table S9: Results of linear mixed-effects model, assessing precipitation and fertilization effect on biomass and biomass sensitivity to precipitation during extreme dry years.

Fixed Effects	Estimate	Standard Error	Degrees of Freedom	t value	P value
Intercept	1.249	0.452	15.3	2.76	0.014
Precipitation	0.406	0.191	12.1	2.13	0.055
Fertilization	0.292	0.223	103.9	1.31	0.192
Precipitation × Fertilization	−0.055	0.094	103.8	−0.58	0.564

Random Effects	Variance	Standard Deviation
Site	0.041	0.20
Block (within site)	0.079	0.28
Year	0.249	0.50
Residual	0.054	0.23

Table S10: Results of paired t-test of differences in the precipitation-biomass correlation (R^2) in control vs. fertilized plots among sites, during extreme dry years.

Metric	Mean difference (Fertilized − Control)	95% Confidence Interval	t value	Degrees of Freedom	P value
R^2	-0.058	0.0096 to 0.107	2.49	31	0.06

Table S11: Results of linear mixed-effects model assessing precipitation and fertilization effect on biomass and biomass sensitivity to precipitation in fenced plots.

Fixed Effects	Estimate	Standard Error	Degrees of Freedom	t value	P value
Intercept	1.37	0.13	828.50	10.23	<0.001
Precipitation	0.42	0.05	1546.40	8.65	<0.001
Fertilization	0.45	0.11	2061.20	4.07	<0.001
Precipitation × Fertilization	−0.112	0.04	2061.60	−2.68	0.007

Random Effects	Variance	Standard Deviation
Block (site)	0.004	0.067
Site	0.039	0.199
Year	0.003	0.056
Residual	0.082	0.287

Table S12: Results of paired t-test of differences in the strength of the precipitation-biomass correlation (R^2) in fenced plots.

Metric	Mean difference (fertilized − unfertilized)	95% Confidence Interval	t value	Degrees of Freedom	P value
R^2	0.0046	-0.031 to 0.040	0.264	29	0.794

Table S13: Results of biomass covariate analysis. Conditional model-averaged predictors of biomass among all study sites. Total candidate models evaluated: 1174. Predictors allowed per model: ≤ 6. Models included in averaging: ΔAICc < 50. Relative importance = summed Akaike weights across the model set.

**Term**	**Estimate**	**Standard Error**	**z value**	**P value**	**Relative importance**
Intercept	1.138	0.110	10.36	<0.001	—
Precipitation	0.400	0.039	10.16	<0.001	**1.000**
Fertilization	0.135	0.015	9.29	<0.001	**1.000**
Light interception	0.375	0.031	12.20	<0.001	**1.000**
Proportion annual lifespan	0.272	0.038	7.20	<0.001	**1.000**
Proportion C_4 × Fertilization	−0.187	0.047	4.01	<0.001	0.980
Aridity index	0.262	0.090	2.89	0.004	0.020
Treatment year	0.006	0.003	2.17	0.060	<0.01
Proportion C_4 photosynthesis	−0.069	0.052	1.33	0.184	1.000
Species richness	0.003	0.002	1.41	0.159	<0.01
Annual proportion × Fertilization	0.044	0.044	1.00	0.319	<0.01
Light interception × Fertilization	0.048	0.041	1.19	0.234	<0.01
Previous growing season precipitation	0.000	0.000	0.29	0.776	<0.01
Precipitation × Fertilization	−0.008	0.040	0.19	0.847	<0.01
Aridity index × Fertilization	0.031	0.029	1.08	0.282	<0.01
Treatment year × Fertilization	0.004	0.003	1.57	0.118	<0.01
Species richness × Fertilization	−0.003	0.002	1.42	0.155	<0.01
Previous growing season precipitation × Fertilization	−0.000	0.000	1.33	0.184	<0.01

Table S14: Results of sensitivity covariate analysis. Conditional model-averaged predictors of biomass sensitivity to precipitation among all study sites. Total candidate models evaluated: 358. Predictors allowed per model: ≤ 6. Models included in averaging: ΔAICc < 10. Relative importance = summed Akaike weights across the model set.

**Term**	**Estimate**	**Standard Error**	**z value**	**P value**	**Relative importance**
Intercept	1.066	0.319	3.34	<0.001	—
Aridity index	−0.801	0.254	3.15	0.002	0.99
Proportion annual lifespan	0.663	0.400	1.66	0.058	0.66
Biomass response ratio	−0.524	0.416	1.26	0.208	0.46
Fertilization	−0.013	0.281	0.05	0.964	0.40
Species richness	−0.013	0.016	0.81	0.416	0.31
Proportion C_4 photosynthesis	−0.258	0.409	0.63	0.528	0.31
Light interception	−0.099	0.363	0.27	0.785	0.25
Biomass response ratio × Fertilization	0.693	0.712	0.97	0.331	0.06
Annual proportion × Fertilization	0.274	0.654	0.42	0.675	0.06
Aridity × Fertilization	0.028	0.403	0.07	0.944	0.08
Species richness × Fertilization	0.009	0.031	0.29	0.769	0.02
Light interception × Fertilization	−0.451	0.685	0.66	0.510	0.02
C_4 proportion × Fertilization	0.019	0.629	0.03	0.976	0.02

Table S15: Results of correlation strength covariate analysis. Conditional model-averaged predictors of the strength of the precipitation-biomass correlation among all study sites. Total candidate models evaluated: 358. Predictors allowed per model: ≤ 6. Models included in averaging: ΔAICc < 10. Relative importance = summed Akaike weights across the model set.

**Term**	**Estimate**	**Standard Error**	**z value**	**P value**	**Relative importance**
Intercept	0.175	0.077	2.27	0.023	—
Aridity index	−0.121	0.058	2.10	0.036	0.81
Proportion annual lifespan	0.176	0.105	1.68	0.093	0.79
Biomass response ratio	−0.132	0.090	1.46	0.143	0.54
Fertilization	−0.018	0.060	0.30	0.764	0.44
Proportion C_4 photosynthesis	0.070	0.101	0.69	0.488	0.34
Light interception	−0.046	0.080	0.58	0.564	0.28
Species richness	0.001	0.004	0.34	0.738	0.24
Annual proportion × Fertilization	0.146	0.144	1.01	0.313	0.11
Aridity × Fertilization	−0.037	0.092	0.40	0.688	0.08
Biomass response ratio × Fertilization	0.070	0.161	0.44	0.662	0.05
Light interception × Fertilization	−0.067	0.153	0.44	0.660	0.02
C_4 proportion × Fertilization	−0.004	0.141	0.03	0.975	0.02
Species richness × Fertilization	−0.003	0.007	0.42	0.672	0.02

Table S16: Author contributions to manuscript

Last Name	First Name	Developed and framed research question(s)	Analyzed data	Contributed to data analyses	Wrote the paper	Contributed to paper writing	Current site-level coordinator	Current network-level coordinator
Slette	Ingrid J.	x	x		x			x
Borer	Elizabeth	x		x		x	x	x
Seabloom	Eric	x		x		x	x	x
Arnillas	Carlos Alberto					x	x	
Bakker	Jonathan D.			x		x	x	
Blumenthal	Dana					x	x	
Catford	Jane A.					x	x	
Collins	Scott					x	x	
Eisenhauer	Nico					x	x	
Fay	Philip A.					x	x	
Garbowski	Magda				x	x		
Haider	Sylvia					x	x	
Harpole	W. Stanley					x	x	
Kirkman	Kevin P.					x	x	
Knops	Johannes M.H.					x	x	
Komatsu	Kimberly J					x	x	
Leakey	Andrew D.B.			x		x		
MacDougall	Andrew S.					x	x	
Martina	Jason P.					x	x	
Power	Sally A.					x	x	
Risch	Anita C.					x	x	
Roscher	Christiane					x	x	
Smith	Melinda D.					x	x	
Tedder	Michelle					x	x	
